# E-cigarette Unit Sales, by Product and Flavor Type — United States, 2014–2020

**DOI:** 10.15585/mmwr.mm6937e2

**Published:** 2020-09-18

**Authors:** Fatma Romeh M. Ali, Megan C. Diaz, Donna Vallone, Michael A. Tynan, Jamie Cordova, Elizabeth L. Seaman, Katrina F. Trivers, Barbara A. Schillo, Brandon Talley, Brian A. King

**Affiliations:** ^1^CDC Foundation, Atlanta, GA; ^2^Truth Initiative, Washington, DC; ^3^Office on Smoking and Health, National Center for Chronic Disease Prevention and Health Promotion, CDC.

Since electronic cigarettes (e-cigarettes) entered the U.S. marketplace in 2007, the landscape has evolved to include different product types (e.g., prefilled cartridge–based and disposable products) and flavored e-liquids (e.g., fruit, candy, mint, menthol, and tobacco flavors), which have contributed to increases in youth use (*1*,*2*). E-cigarettes have been the most commonly used tobacco product among U.S. youths since 2014; in 2019, 27.5% of high school students reported current e-cigarette use (*3*). To assess trends in unit sales of e-cigarettes in the United States by product and flavor type, CDC, CDC Foundation, and Truth Initiative analyzed retail scanner data during September 14, 2014–May 17, 2020, from Information Resources, Inc. (IRI). During this period, total e-cigarette sales increased by 122.2%, from 7.7 million to 17.1 million units per 4-week interval. By product type, the proportion of total sales that was prefilled cartridge products increased during September 2014–August 2019 (47.5% to 89.4%). During August 2019–May 2020, the proportion of total sales that was disposable products increased from 10.3% to 19.8%, while the proportion that was prefilled cartridge products decreased (89.4% to 80.2%). Among prefilled cartridge sales, the proportion of mint sales increased during September 2014–August 2019 (<0.1% to 47.6%); during August 2019–May 2020, mint sales decreased (47.6% to 0.3%), as menthol sales increased (10.7% to 61.8%). Among disposable e-cigarette sales during September 2014–May 2020, the proportion of mint sales increased (<0.1% to 10.5%), although tobacco-flavored (52.2% to 17.2%) and menthol-flavored (30.3% to 10.2%) sales decreased; during the same period, sales of all other flavors combined increased (17.2% to 62.1%). E-cigarette sales increased during 2014–2020, but fluctuations occurred overall and by product and flavor type, which could be attributed to consumer preferences and accessibility. Continued monitoring of e-cigarette sales and use is critical to inform strategies at the national, state, and community levels to minimize the risks of e-cigarettes on individual- and population-level health. As part of a comprehensive approach to prevent and reduce youth e-cigarettes use, such strategies could include those that address youth-appealing product innovations and flavors.

Retail sales data were licensed from IRI, Inc., which included Universal Product Code sales from convenience stores, gas stations, grocery stores, drugstores/pharmacies, mass merchandiser outlets, club stores, dollar stores, and military sales. Sales from the Internet and tobacco-specialty stores, including “vape shops,” were not included. E-cigarette products were categorized as one of the following product types: prefilled cartridge devices, disposable devices, and e-liquids.* E-cigarette accessories and devices sold without e-liquids, which accounted for 9.4% of sales, were excluded. Products with explicit flavor names were categorized as tobacco, menthol, mint, or all other flavors (e.g., fruit, clove/spice, candy/desserts/other sweets, chocolate, alcoholic and nonalcoholic drinks). Ambiguous or concept flavors (e.g., “fusion”) (5.6%) were searched for online and back-coded into one of the four flavor categories. E-cigarette unit sales were standardized and aggregated in 4-week intervals from September 14, 2014, through May 17, 2020^†^ (*4*). Analyses were performed for total unit sales and the proportion of total unit sales by product type and flavor using Stata (version16; StataCorp). Trends during 2014–2020 were analyzed using Joinpoint (version 4.8.0.1; National Cancer Institute), and average 4-week interval percentage change (AIPC) with corresponding 95% confidence intervals (CIs) were calculated. Statistical significance was defined as p<0.05. This study did not involve human subjects, and thus, was not submitted for Institutional Review Board review. 

During September 2014–May 2020, total unit sales increased by 122.2% (p<0.05), from 7.7 million to 17.1 million units per 4-week interval. (AIPC = 1.1; 95% CI = 0.6 to 1.6); however, within the context of this general increase, sales fluctuated (Figure 1). During November 2016–August 2019, sales increased by 294.3%, from 5.6 million to 22.0 million units per period (AIPC = 4.1; 95% CI = 3.2 to 5.1) (p<0.05). During August 2019–February 2020, sales decreased 32.7%, from 22.0 million to 14.8 million units per period (AIPC = −5.1; 95% CI = −7.2 to −2.8) (p<0.05). No significant change in total sales occurred during February–May 2020.

**FIGURE 1:**
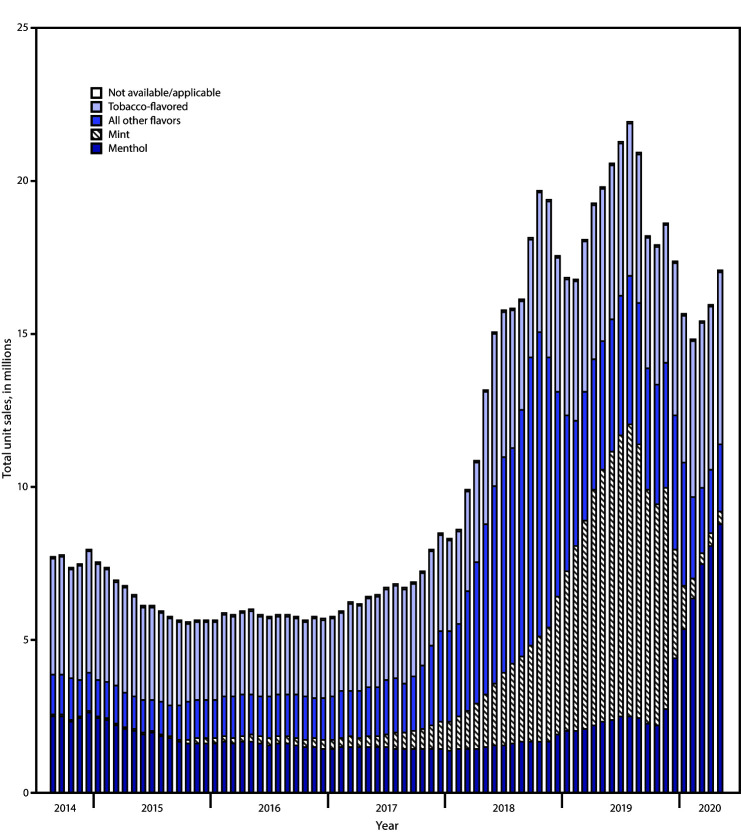
Total e-cigarette unit sales,* by flavor^†^ — United States, September 14, 2014–May 17, 2020^§^ * Retail sales data were obtained from Information Resources, Inc. (IRI) for convenience stores, gas stations, grocery stores, drugstores/pharmacies, mass merchandiser outlets, club stores, dollar stores, and military sales; data from the Internet and vape shops were not collected. ^†^ The “All other flavors” category includes fruit, clove/spice, chocolate, alcoholic drink (such as wine, cognac, or other cocktails), candy/desserts/other sweets, or some other flavor. Unknown flavors were excluded from this figure (<0.1%). ^§^ Each bar in the figure represents a 4-week aggregate interval.

Among total e-cigarette unit sales during September 2014–August 2019, the proportion that were prefilled cartridges increased from 47.5% to 89.4% (AIPC = 1.0) (p<0.05) (Table). The proportion of total sales that were prefilled cartridges decreased thereafter (p<0.05), accounting for 80.2% of total sales in May 2020 (AIPC = −1.3). As the proportion of sales accounted for by prefilled cartridges decreased beginning August 2019, the proportion of sales that were disposable products increased from 10.3% of total sales in August 2019 to 19.8% in May 2020 (AIPC = 7.5) (p<0.05).

**TABLE:** Trends in e-cigarette unit sales, by product and flavor type — United States, September 14, 2014–May 17, 2020

Sales type*	Period	AIPC (95% CI)^†^
**Total sales, by product type**		
Prefilled cartridges^§^	September 2014–August 2019	1.0 (0.8 to 1.2)
	August 2019–May 2020	−1.3 (−1.9 to −0.6)
Disposable devices^¶^	September 2014–August 2019	−2.4 (−3.1 to −1.6)
	August 2019–May 2020	7.5 (4.6 to 10.5)
E-liquid**	September 2014–May 2020	−5.8 (−7.0 to −4.5)
**Total sales, by flavor type**		
Mint	September 2014–August 2019	10.5 (8.1 to 13.0)
	August 2019–May 2020	−28.3 (−36.9 to −18.5)
Menthol	August 2019–May 2020	18.9 (12.5 to 25.7)
Tobacco	August 2019–May 2020	4.6 (2.7 to 6.6)
All other flavors ^† †^	September 2014–October 2018	2.0 (1.3 to 2.7)
	October 2018–May 2020	−5.9 (−8.3 to −3.4)
**Prefilled cartridge sales, by flavor type**		
Mint	September 2014–August 2019	14.1 (8.5 to 20.1)
	August 2019–May 2020	−42.3 (−54.6 to −26.7)
Menthol	August 2019–May 2020	22.3 (14.9 to 30.1)
Tobacco	August 2019–May 2020	6.1 (3.6 to 8.7)
All other flavors	September 2014–October 2018	3.3 (2.3 to 4.2)
	October 2018–May 2020	−18.1 (−28.6 to −6.0)
**Disposable sales, by flavor type**		
Mint	September 2014–May 2020	7.4 (4.7 to 10.1)
Menthol	September 2014–May 2020	−1.4 (−2.5 to −0.3)
Tobacco	September 2014–May 2020	−1.5 (−2.1 to −0.9)
All other flavors	September 2014–May 2020	1.6 (1.3 to 1.9)
**E-liquid sales, by flavor type**		
Mint	September 2014–May 2020	−3.5 (−4.9 to −2.2)
Menthol	September 2014–May 2020	—^§§^
Tobacco	September 2014–May 2020	−4.5 (−6.7 to −2.3)
All other flavors	September 2014–May 2020	−4.2 (−5.9 to −2.4)

**Abbreviations:** AIPC = average 4-week interval percentage change; CI = confidence interval.

* Retail sales data were obtained from Information Resources, Inc. (IRI) for convenience stores, gas stations, grocery stores, drug stores/pharmacies, mass merchandiser outlets, club stores, dollar stores, and military sales; data from the Internet and vape shops were not collected.

^†^ AIPC (CI) calculated using Joinpoint (version 4.8.0.1; National Cancer Institute).

^§^ Prefilled cartridges include tanks, cartridges, and pods used in rechargeable and reusable e-cigarette device; the cartridges are not intended to be refilled after the liquid has been depleted. Unit sales were standardized to reflect the most common package size for each product type; a standardized unit was equal to five prefilled cartridges.

^¶^ Disposable devices include nonrechargeable and nonreusable e-cigarette devices that are not intended to be refilled with e-liquid after being depleted; the device is disposed of once the e-liquid has been consumed. Unit sales were standardized to reflect the most common package size for each product type; a standardized unit was equal to 1 disposable device.

** E-liquids are containers of the liquid used in e-cigarette devices, which typically contains a humectant (e.g., propylene glycol), nicotine, and flavoring.

^††^ The “All other flavors” category includes fruit, clove/spice, chocolate, alcoholic drink (such as wine, cognac, or other cocktails), candy/desserts/other sweets, or some other flavor. Unknown flavors were excluded from this figure (<0.1%).

^§§^ The dash indicates that Joinpoint regression could not be conducted because of small sales values.

Among total e-cigarette unit sales during September 2014–August 2019, the proportion accounted for by mint products increased from 0.01% to 43.4% (AIPC = 10.5) (p<0.05) (Figure 1). During August 2019–May 2020, although mint sales declined from 43.4% to 2.3% of total e-cigarette sales (AIPC = −28.3), the proportion of menthol sales increased from 11.4% to 51.6% of total sales (AIPC = 18.9), and tobacco-flavored sales increased from 23.0% to 33.1% of total sales (AIPC = 4.6). During September 2014–October 2018, sales of all other flavored e-cigarettes increased from 17.6% to 52.4% of total sales (AIPC = 2.0) (p<0.05); however, sales of all other flavored e-cigarettes declined thereafter, from 52.4% to 12.8% of total sales by May 2020 (AIPC = −5.9) (p<0.05).

Among prefilled cartridge sales during September 2014–August 2019, the percentage that were mint increased from <0.1% to 47.6% (AIPC = 14.1) (p<0.05) (Figure 2). During August 2019–May 2020, although the mint sales declined from 47.6% to 0.3% of all prefilled cartridge sales (AIPC = −42.3), the proportion of menthol sales increased from 10.7% to 61.8% (AIPC = 22.3), and the percentage of tobacco-flavored sales increased from 22.8% to 37.1% (AIPC = 6.1). During September 2014–October 2018, sales of all other flavors increased from 12.9% to 54.4% of prefilled cartridge sales (AIPC = 3.3) (p<0.05); however, sales of these products declined thereafter to 0.8% of all prefilled cartridge sales by May 2020 (AIPC = −18.1) (p<0.05).

**FIGURE 2:**
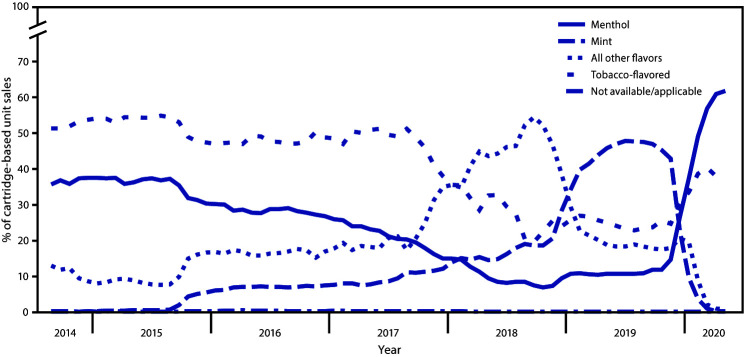
Percentage of prefilled cartridge* e-cigarette unit sales,^†^ by flavor^§^— United States, September 14, 2014–May 17, 2020 * Prefilled cartridges include tanks, cartridges, and pods used in rechargeable and reusable e-cigarette device; the cartridges are not intended to be refilled after the liquid has been depleted. Unit sales were standardized to reflect the most common package size for each product type; a standardized unit was equal to 5 prefilled cartridges. ^†^ Retail sales data were obtained from Information Resources, Inc. (IRI) for convenience stores, gas stations, grocery stores, drugstores/pharmacies, mass merchandiser outlets, club stores, dollar stores, and military sales; data from the Internet and vape shops were not collected. ^§^ The “All other flavors” category includes fruit, clove/spice, chocolate, alcoholic drink (such as wine, cognac, or other cocktails), candy/desserts/other sweets, or some other flavor. Unknown flavors were excluded from this figure (<0.1%).

Among disposable e-cigarette sales during September 2014–May 2020, the percentage of sales of tobacco-flavored and menthol-flavored products decreased; sales of tobacco-flavored e-cigarettes accounted for 17.2% and menthol-flavored accounted for 10.2% of all disposable e-cigarette sales in May 2020, (p<0.05). (Figure 3). During the same period, mint-flavored sales increased from <0.1% to 10.5% of all disposable e-cigarette sales (AIPC = 7.4), and the proportion of all other flavors increased from 17.2% to 62.1% (AIPC = 1.6).

**FIGURE 3:**
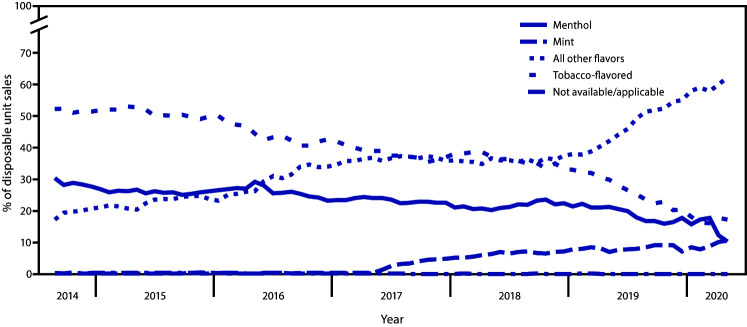
Percentage of disposable e-cigarette* unit sales,^†^ by flavor^§^— United States, September 14, 2014–May 17, 2020 * Disposable devices include nonrechargeable and nonreusable e-cigarette devices that are not intended to be refilled with e-liquid after being depleted; the device is disposed of once the e-liquid has been consumed. Unit sales were standardized to reflect the most common package size for each product type; a standardized unit was equal to 1 disposable device. ^†^ Retail sales data were obtained from Information Resources, Inc. (IRI) for convenience stores, gas stations, grocery stores, drugstores/pharmacies, mass merchandiser outlets, club stores, dollar stores, and military sales; data from the Internet and vape shops were not collected. ^§^ The “All other flavors” category includes fruit, clove/spice, chocolate, alcoholic drink (such as wine, cognac, or other cocktails), candy/desserts/other sweets, or some other flavor. Unknown flavors were excluded from this figure (<0.1%).

## Discussion

During November 2016–August 2019, total e-cigarette unit sales in the U.S. increased nearly 300%. Although prefilled cartridges remained the leading product type sold, disposable sales increased beginning in August 2019, reaching 19.8% of total sales by May 2020. Among prefilled cartridge sales, the proportion of mint-flavored products declined beginning in August 2019; by May 2020, menthol (61.8%) and tobacco (37.1%) flavors dominated the market. Among disposable e-cigarette sales, tobacco-flavored and menthol-flavored sales decreased during September 2014–May 2020; during the same period, the proportion of sales that were mint and all other flavors increased, with mint reaching 10.5% and all other flavors reaching 62.1% of total sales by May 2020. Continued monitoring of e-cigarette sales could inform strategies to reduce use among U.S. youths, including strategies that address youth-appealing product innovations and flavors (*1,2*).

The increase in total e-cigarette sales that occurred during November 2016–August 2019 was driven by sales of prefilled cartridges, which made up nearly 90% of the market by August 2019. Previous research indicates this increase in total sales was primarily driven by JUUL (*5*), a prefilled cartridge-based e-cigarette that accounted for approximately 75% of total U.S. e-cigarette sales by December 2018.^§^ The rise in JUUL sales occurred during the same period as when youth e-cigarette use increased considerably; during 2017–2018, current e-cigarette use increased 78% among U.S. high school students and 48% among middle school students (*6*). The decline in total e-cigarettes sales during August 2019–February 2020 might be attributable, in part, to shifts in consumer behaviors following the national outbreak of e-cigarette, or vaping, product use-associated lung injury (EVALI) (*7*).

Among prefilled cartridge e-cigarettes, sales of mint and other flavors declined beginning in August 2019, after which menthol and tobacco-flavored sales increased considerably. During the same period, overall disposable e-cigarette sales increased, particularly mint and other flavored (excluding menthol or tobacco) products. Flavored e-cigarette sales patterns by product type are likely influenced by multiple factors. For example, JUUL voluntarily removed mango, creme, fruit, and cucumber flavored cartridges from retail stores (November 2018) and online (October 2019)^¶^ and removed mint-flavored cartridges entirely from the market in November 2019.** Moreover, on January 2, 2020, the Food and Drug Administration (FDA) finalized an enforcement policy that prohibits the sale of prefilled cartridge e-cigarettes in any flavor other than tobacco or menthol.^††^

The findings in this report are subject to at least three limitations. First, sales data did not include purchases from the Internet or “vape shops,” which accounted for approximately one half of U.S. e-cigarette sales in 2019;^§§^ a data source for Internet and “vape shop” sales does not currently exist. Second, the study could not assess purchaser age. These sales could reflect products purchased by adults or those obtained directly or indirectly by youths; however, three quarters of youths who use JUUL, the mostly commonly sold e-cigarette brand in the United States, reported obtaining it from a physical retail location.^¶¶^ Finally, ambiguous or concept flavors were back-coded using online searches and might be subject to misclassification; however, this only applied to 5.6% of total sales.

Youth use of tobacco products in any form, including e-cigarettes, is unsafe (*1,2*). In the U.S., e-cigarette use is markedly higher among youths than adults; in 2018, current use of e-cigarettes was 20.8% (past 30-day use) among high school students, 7.6% (everyday/someday use) among adults aged 18–24 years, and 3.2% (everyday/someday use) among adults aged ≥18 years (*6**,**8*). In addition to regulation of the manufacturing, marketing, and sale of e-cigarettes by FDA,*** strategies to reduce e-cigarette use among youths include increasing price, implementing comprehensive smoke-free policies that include e-cigarettes, restricting youths’ access to e-cigarettes in retail settings, licensing retailers, developing educational initiatives targeting youths, curbing youth-appealing advertising and marketing, and implementing strategies to reduce youth access to flavored tobacco products (*1*,*2*,*9*).

**Summary**

**What is already known about this topic?**

Since electronic cigarettes (e-cigarettes) entered the U.S. marketplace in 2007, the landscape has evolved to include disposable e-cigarettes and rechargeable e-cigarettes with prefilled cartridges and flavored e-liquids (e.g., fruit, candy, and mint).

**What is added by this report?**

During September 2014–May 2020, e-cigarette sales increased by 122.2%. Sales of prefilled cartridges increased during September 2014–August 2019; since then, sales of disposable products have increased. Prefilled mint cartridge e-cigarette sales increased from September 2014 to August 2019, then decreased, as menthol sales increased during August 2019–May 2020.

**What are the implications for public health practice?**

Continued monitoring of e-cigarette sales and use is critical to inform strategies to minimize risks. As part of a comprehensive approach, such strategies could include those that address youth-appealing product innovations and flavors.

## Footnotes

* Prefilled cartridges include tanks, cartridges, and pods used in rechargeable and reusable e-cigarette device; the cartridges are not intended to be refilled after the liquid has been depleted. Disposable devices include nonrechargeable and nonreusable e-cigarette devices that are not intended to be refilled with e-liquid after being depleted; the device is disposed of once the e-liquid has been consumed. E-liquids are containers of the liquid used in e-cigarette devices, which typically contains a humectant (e.g., propylene glycol), nicotine, and flavoring.

† Consistent with previous studies, unit sales were standardized to reflect the most common package size for each product type. A standardized unit was equal to five prefilled cartridges, one disposable device, or one e-liquid bottle.

§ https://truthinitiative.org/research-resources/emerging-tobacco-products/behind-explosive-growth-juul.

¶ https://www.juullabs.com/juul-labs-suspends-sale-of-non-tobacco-non-menthol-based-flavors-in-the-u-s/.

** https://www.juullabs.com/juul-labs-stops-the-sale-of-mint-juulpods-in-the-united-states/.

††
https://www.fda.gov/news-events/press-announcements/fda-finalizes-enforcement-policy-unauthorized-flavored-cartridge-based-e-cigarettes-appeal-children .

§§
http://www.natocentral.org/uploads/Wall_Street_Update_Slide_Deck_February_2019.pdf .

¶¶ https://truthinitiative.org/research-resources/emerging-tobacco-products/where-are-kids-getting-juul.

*** https://pubmed.ncbi.nlm.nih.gov/27192730/.
